# The DigH glycosyl hydrolase is conditionally required for daughter cell separation in *Escherichia coli*

**DOI:** 10.1128/jb.00068-25

**Published:** 2025-06-10

**Authors:** Joseph C. Bryant, Emily J. Robbs, Alongkorn Kurilung, Brittney A. Dinkel, Intawat Nookaew, Matthew A. Jorgenson

**Affiliations:** 1 Department of Microbiology and Immunology, University of Arkansas for Medical Sciences 318223, Little Rock, Arkansas, USA; 2 Department of Biomedical Informatics, University of Arkansas for Medical Sciences 12215, Little Rock, Arkansas, USA; 3 School of Science, Buena Vista University 7707, Storm Lake, Iowa, USA; Queen Mary University of London, London, United Kingdom

**Keywords:** peptidoglycan, lytic transglycosylase, glycosyl hydrolase, denuded glycans, cell separation

## Abstract

The peptidoglycan (PG) cell wall is a mesh-like layer that shapes bacteria and protects against osmotically induced lysis. PG is composed of glycan strands and peptide chains that link together to form a continuous layer that surrounds the cell. PG hydrolases are required for cell wall maturation, and many are employed during cell separation. During cell division, amidases remove peptides from the glycan backbone, and the resulting denuded glycans (dnGs) are degraded by lytic transglycosylases (LTs). The gram-negative bacterium *Escherichia coli* encodes eight functional LTs (*mltA-G* and *slt*) and one putative LT (*rlpA*), and a mutant strain lacking six (Δ*mltACDE*Δ*slt*Δ*rlpA*), which we refer to as ΔLT cells, accumulates dnGs and produces short chains of cells. A morphological suppressor of the ΔLT chaining defect was isolated, and deletion analysis indicated that suppression relied primarily on increased activity of DigH, a denuded-specific hydrolase that accumulates at the midcell during cell division. Further analyses revealed that DigH is critical for cell separation in ΔLT but not wild-type cells and that dnGs accumulate even more in ΔLT cells when DigH is absent. Thus, DigH is a denuded-specific hydrolase that is conditionally required for cell separation in *E. coli*. Altogether, our findings deepen our understanding of the specific cellular function of DigH and of PG maturation in *E. coli*.

**IMPORTANCE:**

Most bacteria are surrounded by an essential polymer known as the peptidoglycan cell wall. During cell division, a transient form of peptidoglycan is generated between the developing daughter cells that must be cleaved so that cells can separate. Here, we show that the DigH hydrolase is conditionally required for cell separation when this transient cell wall structure accumulates in the gram-negative bacterium *Escherichia coli*. These findings deepen our understanding of how the peptidoglycan layer is remodeled during cell division.

## INTRODUCTION

Bacteria are found in a wide array of shapes and sizes (1) and, for most, cell shape (also referred to as morphology) is generated by the peptidoglycan (PG) cell wall. PG is a heteropolymer composed of glycan strands linked by short peptides. The glycan strands are made from a repeating disaccharide of *N*-acetylglucosamine (GlcNAc) and *N*-acetylmuramic acid (MurNAc) (2). Adjacent glycan strands are connected by short peptides to form a continuous structure (referred to as the sacculus) that shapes the cell and protects against turgor (3). The PG layer is a dynamic structure that is selectively cleaved during growth and division by PG hydrolases to allow for the insertion of new material. PG hydrolases cleave nearly every linkage in PG and can be broadly classified as peptidases or glycosidases (4). Peptidases cleave the stem peptide and include amidases (release stem peptides from glycan chains), carboxypeptidases (trim the ends of stem peptides), and endopeptidases (cleave cross-links between stem peptides) (5). Glycosidases cleave linkages within glycan chains and include glucosaminidases (hydrolyze the glycosidic bond between GlcNAc and MurNAc), muramidases (hydrolyze the glycosidic bond between MurNAc and GlcNAc), and lytic transglycosylases (non-hydrolytically cleave the glycosidic bond between MurNAc and GlcNAc) (5).

Determining the physiological roles that PG hydrolases (and their activators) play during cell wall maturation is usually accomplished by interpreting a combination of mutant phenotypes and biochemical assays using purified PG substrates. However, PG hydrolases in bacteria are highly redundant, and individual mutants are often indistinguishable from wild-type cells (6). This redundancy helps explain why functional data is missing for many PG hydrolases (7). The most direct way to address functional redundancy is to mutate or delete multiple PG hydrolases at once and then test for mutant phenotypes (which are often growth or morphological). Using this approach in *E. coli*, combination mutants have revealed the importance of amidases for cell division (8–10), endopeptidases for PG expansion (11), and carboxypeptidases for morphological maintenance (12, 13). Functional redundancy also exists for lytic transglycosylases (LTs). The *E. coli* genome encodes seven membrane-bound LTs (*mltA*, *mltB*, *mltC*, *mltD*, *mltE*, *mltF*, and *mltG*), as well as one soluble periplasmic LT (*slt*). Mutants lacking various combinations of these LTs grow as short chains (approximately 3–8 cells), with individual cell units within the chain being somewhat shorter than normal (14, 15). Chaining has also been observed for LT combination mutants in *Salmonella enterica* (16) and *Vibrio cholerae* (17). Collectively, these findings indicate that LTs are important for both growth and division.

Previously, we engineered a mutant of *E. coli* deleted for five LTs (Δ*mltACDE*Δ*slt*) and *rlpA* (15), which appears to have lost LT activity (18). Hereafter, we refer to this strain as “ΔLT.” During cell division, amidases remove stem peptides from glycan chains at the septum, and the resulting peptide-free (denuded) glycans are degraded by LTs. ΔLT cells accumulate denuded glycans (dnGs) and grow as short chains, most likely due to glycan strands being shared between the developing daughter cells (15). While characterizing ΔLT cells, we isolated a spontaneous suppressor of this strain background that grows as normal rods. Further analysis indicated that the suppression phenotype was driven primarily by increased activity of DigH, a denuded-specific hydrolase (with either glucosaminidase or muramidase activity) that preferentially accumulates at the midcell of dividing cells (19). However, since no morphological phenotypes have been reported for a *digH* deletion, we decided to investigate the function of DigH in the context of ΔLT cells. Here, we present morphological evidence that DigH activity is functionally redundant with one or more LT and that DigH is conditionally required for cell separation in *E. coli*. Altogether, our findings help clarify the specific functional role of DigH and, more broadly, our understanding of PG degradation at the septum.

## RESULTS

### Selection for ΔLT suppressor mutations

**Fig 1:**
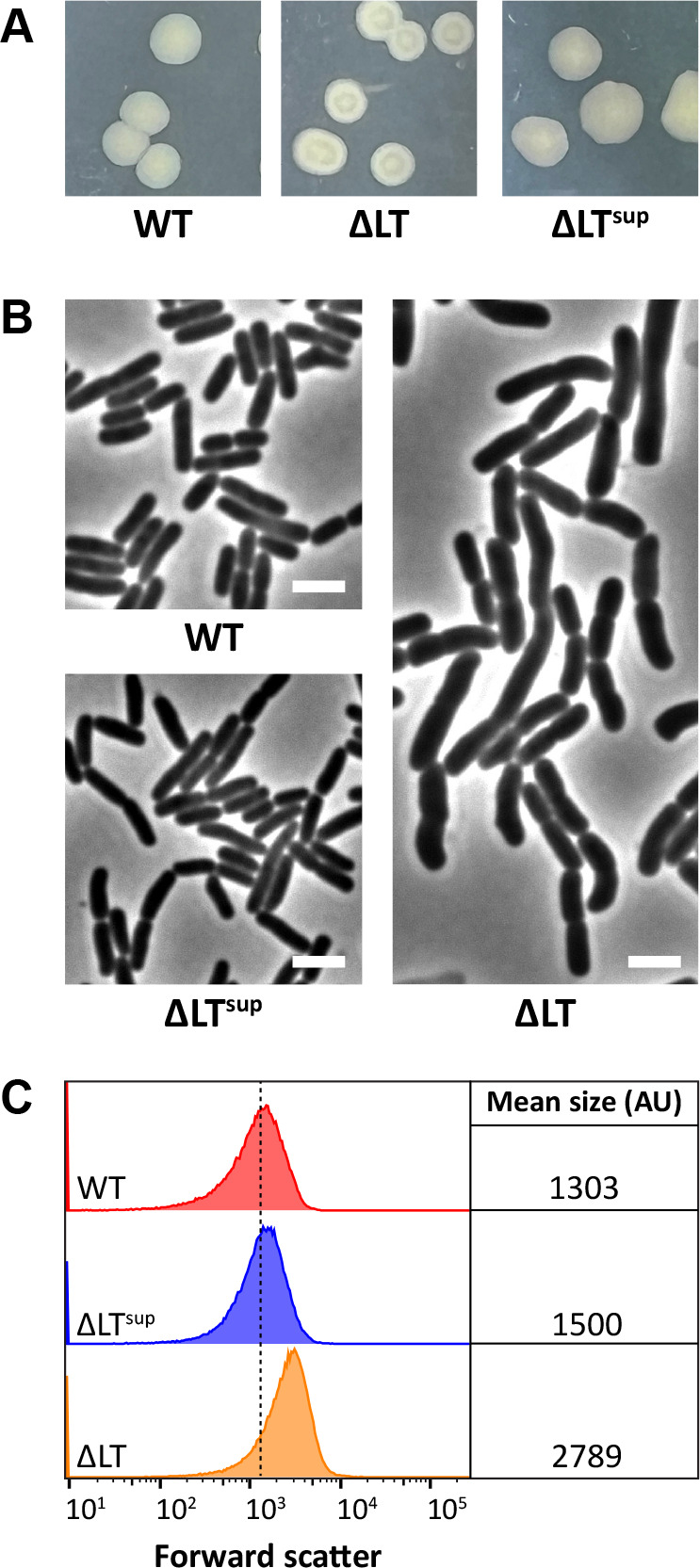
Growth and morphological phenotypes of a ΔLT suppressor. (**A**) Colony phenotypes of cells with the indicated genotypes. We note that ΔLT colonies are mucoid. (**B**) Cells with the indicated genotypes were grown in LB at 37°C until the culture reached an OD_600_ of ~0.4–0.5. The cells were then photographed by phase-contrast microscopy. The white bar represents 3 µm. (**C**) Live cells from panel B were also examined by flow cytometry. Histograms of the forward scatter area from 100,000 events (cells) are shown. The mean cell size of the wild-type is represented by the dashed line and is expressed in arbitrary units (AU). Data are representative of two independent experiments. The strains shown are MAJ1 (WT), MAJ718 (ΔLT), and MAJ1075 (ΔLT^sup^).

Previously, we transformed ΔLT cells with plasmid pMAJ77, a derivative of pDSW209 that expresses a GFP fusion to the cell division protein YtfB (20). Since YtfB contains a LysM-like domain (i.e., OapA domain) and since LysM domains bind PG and localize to septal regions (21), we reasoned that YtfB localization would increase in cells that accumulate dnGs (which we confirmed) (20). While isolating ΔLT/pMAJ77 transformants, we observed that ΔLT/pMAJ77 cells form mucoid colonies with depressions when plated on LB media containing ampicillin (to select for pMAJ77) at 37°C (not shown). Curiously, the transformation also yielded several non-mucoid colonies. Since mutations that activate the Rcs stress response induce mucoidy (due to the production of colanic acid) (22), we reasoned the non-mucoid colonies were ΔLT suppressors. Ampicillin was not required to reverse mucoidy as similar colony phenotypes were obtained for ΔLT cells (without plasmid) plated on LB media at 37°C (Fig. 1A). We observed that approximately 2% of colonies plated on LB were non-mucoid; one of these suppressors (hereafter referred to as ΔLT^sup^ cells) was chosen for further analysis. We note that ΔLT suppressors were originally obtained by growing ΔLT cells overnight in a New Brunswick Model G76 Water Bath Shaker set at 37°C (water temperature was verified with a thermometer). Overnight cultures were then plated onto LB agar and incubated at 37°C overnight. Interestingly, later efforts to obtain ΔLT suppressors in a New Brunswick C76 Shaker required growing ΔLT cells at 38.5°C and then plating onto LB plates at 37°C. Upon closer inspection, we noticed that the heating element in the G76 shaker (but not in the C76 shaker) is exposed and located directly beneath the metal plate that holds the culture tube rack. Thus, high temperature likely selected for ΔLT suppression. Since ΔLT cells produce short chains of unseparated cells that are also enlarged (15), we examined ΔLT^sup^ cells for shape suppression by microscopy and flow cytometry. As shown in Fig. 1B, ΔLT^sup^ cells were rod-shaped (i.e., wild type). Similarly, the forward scatter distribution of ΔLT^sup^ cells was shifted to the left, confirming that the cells had returned to wild-type size (Fig. 1C).

### Disrupting the Prc-NlpI proteolytic system suppresses the shape defect of ΔLT cells

**Fig 2:**
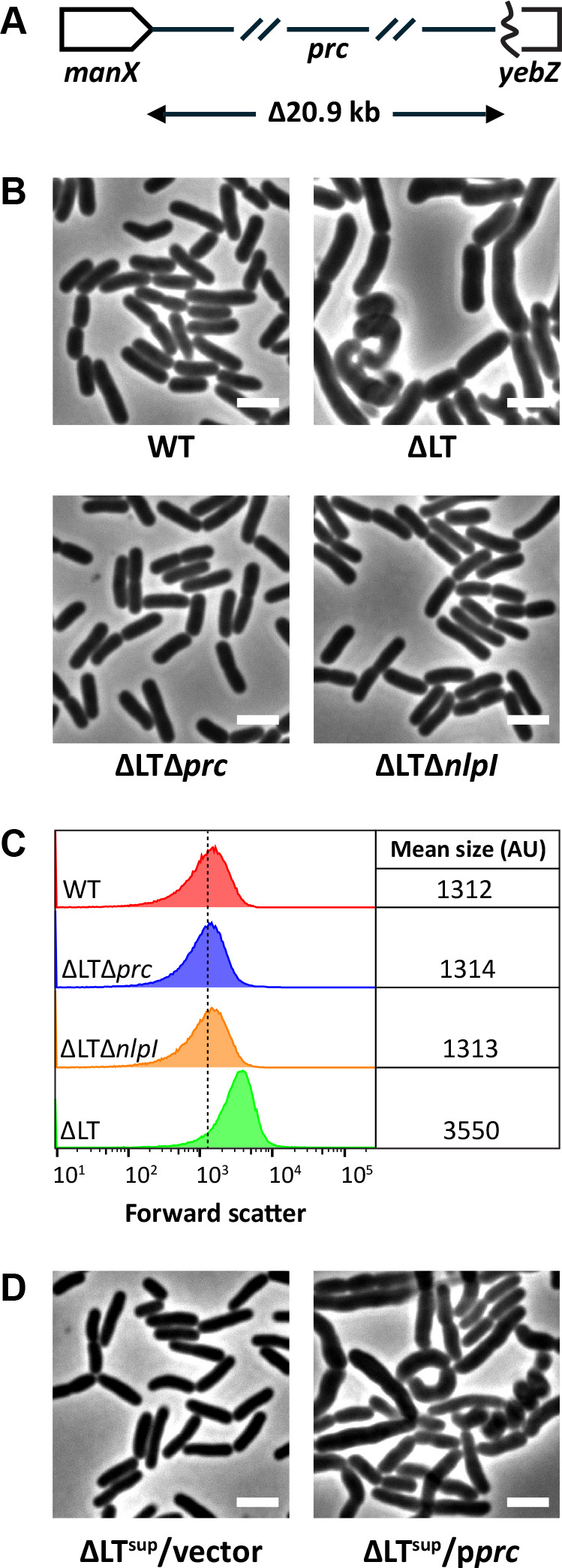
Disrupting the NlpI-Prc system reverses the shape defect of ΔLT cells. (**A**) The ΔLT^sup^ deletion encompasses the genomic region between *manX* and *yebZ* (NC_000913.3 genomic region 1,903,026-1,924,018). The identities of the genes deleted in ΔLT^sup^ cells are listed in Table 1. (**B**) Cells with the indicated genotypes were grown in LB at 37°C until the culture reached an OD_600_ of ~0.4–0.5. The cells were then photographed by phase-contrast microscopy. Bar, 3 µm. (**C**) Live cells from panel B were also examined by flow cytometry. Histograms of the forward scatter area from 100,000 events (cells) are shown. The mean cell size of the wild-type is represented by the dashed line and is expressed in arbitrary units (AU). (**D**) Micrographs of ΔLT^sup^ cells containing vector or plasmid p*prc*. Cells were grown at 37°C in LB (without induction) until the culture reached an OD_600_ of 0.4–0.5. The cells were then photographed by phase-contrast microscopy. The white bar represents 3 µm. Data are representative of two independent experiments. The strains shown are MAJ1 (WT), MAJ718 (ΔLT), MAJ1801 (ΔLTΔ*prc*), MAJ1780 (ΔLTΔ*nlpI*), MAJ1895 (ΔLT^sup^/vector), and MAJ1897 (ΔLT^sup^/p*prc*).

We next used whole-genome sequencing to determine if there were one or more suppressing mutation(s) in ΔLT^sup^ cells. To do this, we employed a hybrid approach using Illumina (short read) and Nanopore (long read) sequencing. Subsequent sequence analysis revealed a 20,992 bp deletion (NC_000913.3 genomic region 1903026-1924018) in ΔLT^sup^ cells, which we confirmed by PCR and Sanger sequencing; no other mutations were identified. The deletion in ΔLT^sup^ cells begins immediately downstream of *manX* and extends into the 3′ end of *yebZ* (Fig. 2A) and encompasses at least 26 open reading frames (Table 1). Among the genes deleted in the ΔLT^sup^ strain was *prc*, which encodes a periplasmic protease that is known to cleave cell wall synthases (23–25) and hydrolases (19, 26, 27). To our knowledge, no other factors directly or indirectly connected to cell wall metabolism are present in the ΔLT^sup^ deletion (Table 1). Though most chromosomal rearrangements occur by recombination between homologous sequences including transposons, insertion sequence (IS) elements, and prophage remnants (28), we did not detect these sorts of sequences in the region deleted in ΔLT^sup^ cells. However, we observed sequences within the breakpoint (8–9 bps in length) that were also present but inverted in *prc* (Fig. S1). The identification of inverted repeats suggests that the ΔLT^sup^ deletion may have arisen from two chromosomal rearrangements, i.e., inversion followed by deletion. We are currently investigating the importance of these repeated sequences.

**TABLE 1:** Genes deleted in ΔLT^sup^ cells

Gene name	Genomic position^*a*^	Product
*manY*	1,903,082 → 1,903,882	Mannose-specific PTS enzyme IIC component
*manZ*	1,903,895 → 1,904,746	Mannose-specific PTS enzyme IID component
*yobD*	1,904,801 → 1,905,259	Protein of unknown function
*mntP*	1,905,688 → 1,906,254	Manganese exporter
*rlmA*	1,906,251 ← 1,907,060	rRNA large subunit methyltransferase A
*cspC*	1,907,226 ← 1,907,435	Transcription antiterminator
*yobF*	1,907,448 ← 1,907,591	Protein of unknown function
*yebO*	1,908,261 ← 1,908,548	Protein of unknown function
*mgrB*	1,908,623 ← 1,908,766	PhoQ kinase inhibitor
*yobH*	1,908,925 → 1,909,164	Protein of unknown function
*kdgR*	1,909,308 ← 1,910,099	Transcriptional repressor
*yebQ*	1,910,276 → 1,911,649	Putative efflux pump
*htpX*	1,911,695 ← 1,912,576	Protease
*prc*	1,912,768 ← 1,914,816	Periplasmic protease
*proQ*	1,914,836 ← 1,915,534	RNA chaperone
*msrC*	1,915,631 ← 1,916,128	Methionine-(R)-sulfoxide reductase
*letA*	1,916,258 → 1,917,541	Integral inner membrane transport protein
*letB*	1,917,510 → 1,920,143	Lipophilic envelope spanning tunnel
*rsmF*	1,920,223 → 1,921,662	16S rRNA methyltransferase
*yebV*	1,921,780 → 1,922,016	Protein of unknown function
*yebW*	1,922,121 → 1,922,312	Protein of unknown function
*pphA*	1,922,313 ← 1,922,969	Phosphoprotein phosphatase
*ryeA*	1,923,066 → 1,923,337	Small antisense RNA
*sdsR*	1,923,104 ← 1,923,207	Small regulatory RNA
*yebY*	1,923,365 ← 1,923,706	Protein of unknown function
*yebZ* ^*b*^	1,923,719 ← 1,924,591	Putative copper importer

^*a*^ Positions correspond to NCBI Reference Sequence NC_000913.3.

^*b*^ *yebZ* is truncated (positions 1,923,719 ← 1,924,018) in ΔLT^sup^ cells.

At this point, we reasoned that the loss of Prc suppressed chaining in ΔLT^sup^ cells. To confirm that shape suppression in ΔLT^sup^ cells was due to *prc* inactivation alone, we deleted *prc* from ΔLT cells and examined the mutant derivative for shape suppression. As shown in Fig. 2B and C, deleting *prc* reversed the morphological defects produced by ΔLT cells. Shape suppression was reversed by expressing *prc* from a plasmid in ΔLT^sup^ cells (Fig. 2D), further confirming that loss of Prc underlined shape suppression in ΔLT^sup^ cells. We note that *prc* was cloned under strict control of IPTG (i.e., plasmid pDSW206) to circumvent the selection of inactive *prc* variants, which were repeatedly isolated when attempting to use our standard IPTG-inducible expression vector pDSW204 (29). We note that plasmid pDSW206 is pDSW204 with promoter down mutations (29). Since the adapter protein NlpI helps coordinate Prc proteolytic activity (26, 30), we reasoned that deleting *nlpI* would also suppress shape defects in ΔLT cells. As expected, ΔLTΔ*nlpI* cells mirrored the shape and size of ΔLTΔ*prc* cells (Fig. 2B and C). In summary, our results demonstrate that disrupting the Prc-NlpI complex suppresses morphological defects produced by ΔLT cells.

### Multiple Prc-regulated hydrolases are required to maintain morphology in ΔLTΔ*prc* cells

**Fig 3:**
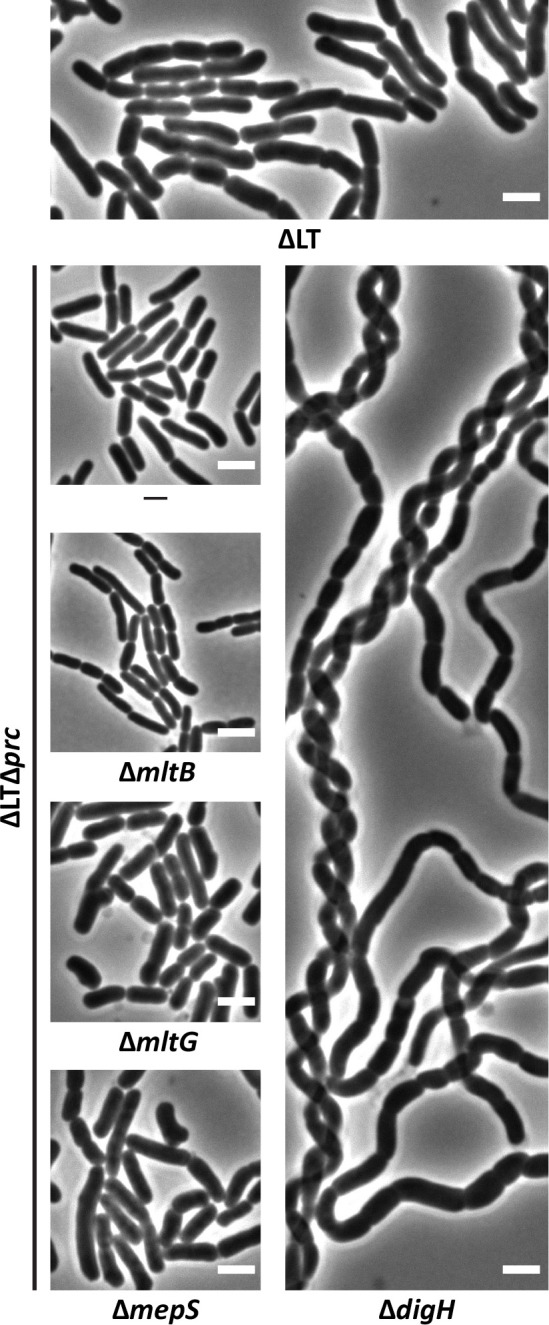
Prc protease substrates are required for shape suppression in ΔLTΔ*prc* cells. Cells with the indicated genotypes were grown in LB at 37°C until the culture reached an OD_600_ of ~0.4–0.5. The cells were then photographed by phase-contrast microscopy. Bar, 3 µm. Data are representative of two independent experiments. The strains shown are MAJ718 (ΔLT), MAJ1801 (ΔLTΔ*prc*), MAJ1834 (ΔLTΔ*prc*Δ*mltB*), MAJ1813 (ΔLTΔ*prc*Δ*mltG*), MAJ1807 (ΔLTΔ*prc*Δ*mepS*), and MAJ1812 (ΔLTΔ*prc*Δ*digH*).

**Fig 4:**
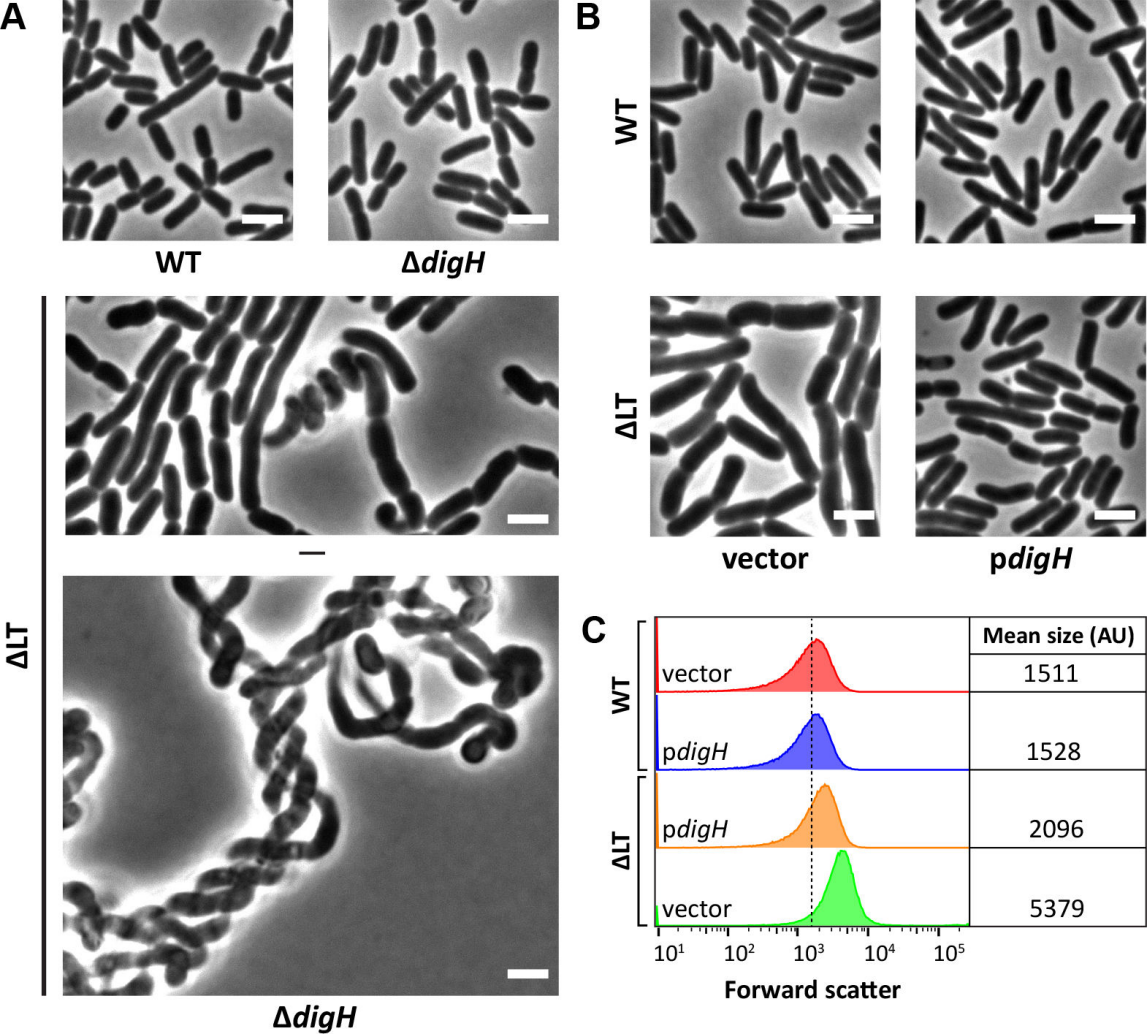
DigH promotes cell separation in cells that accumulate denuded glycans. (**A**) Cells with the indicated genotypes were grown in LB at 37°C until the culture reached an OD_600_ of ~0.4–0.5, with the exception of ΔLTΔ*digH* cells, which were imaged after overnight growth at 37°C. The cells were then photographed by phase-contrast microscopy. The white bar represents 3 µm. (**B**) Micrographs of ΔLT cells containing vector or p*digH*. Cells were grown at 37°C in LB containing 25 µM IPTG. The cells were then photographed by phase-contrast microscopy. Bar, 3 µm. (**C**) Live cells from panel B were also examined by flow cytometry. Histograms of the forward scatter area from 100,000 events (cells) are shown. The mean cell size of the wild-type is represented by the dashed line and is expressed in arbitrary units (AU). Data are representative of two independent experiments. The strains shown are MAJ1 (WT), MAJ1814 (Δ*digH*), MAJ718 (ΔLT), MAJ1815 (ΔLTΔ*digH*), MAJ286 (WT/vector), MAJ1934 (WT/p*digH*), MAJ1808 (ΔLT/vector), and MAJ1830 (ΔLT/p*digH*).

In addition to selectively degrading proteins with nonpolar C-termini (31), Prc negatively regulates the post-translational stability of several cell wall hydrolases in *E. coli,* including the MepS endopeptidase (26), the MltB and MltG lytic transglycosylases (19, 27), and the DigH glycosyl hydrolase, which specifically degrades dnGs (i.e., the PG structure that accumulates in ΔLT cells) (19). Thus, we reasoned that increased cell wall hydrolase activity in one or more of these enzymes was responsible for shape suppression in ΔLT^sup^/ΔLTΔ*prc* cells. To determine which Prc substrates were required to maintain rod shape, we individually deleted *mepS*, *mltB*, *mltG*, and *digH* from ΔLTΔ*prc* cells and assayed the resulting mutant derivatives for shape reversion (i.e., increased chaining and other morphological defects). As shown in Fig. 3, deleting *mltB* from ΔLTΔ*prc* cells induced mild chaining, while deleting *mltG* and, to a greater extent, *mepS* produced shape defects (i.e., widening, branching, and chaining) reminiscent of the ΔLT parent. Notably, deleting *digH* from ΔLTΔ*prc* cells produced the most severe synthetic effect. ΔLTΔ*prc*Δ*digH* cells grew as extremely long chains (Fig. 3), indicating a complete block in cell separation. We also note that ΔLTΔ*prc*Δ*digH* cells grew poorly and flocculated when cultured in liquid media (not shown). Mutants individually deleted for *mepS*, *mltB*, *mltG*, and *digH* are shown in Fig. S2; Fig. 4A. We note that cells lacking Prc, MepS, NlpI, or MltG produced mild shape defects (Fig. S2). Collectively, these findings suggest that shape suppression in ΔLTΔ*prc* cells is likely achieved by the activities of multiple Prc-regulated hydrolases. Since deleting *digH* produced the most severe effect on ΔLTΔ*prc* cells, we sought to investigate the connection to DigH further.

### DigH promotes cell separation in cells that accumulate denuded glycans

DigH was originally identified as a multicopy suppressor of Δ*tol-pal* phenotypes. Similar to ΔLT cells, Δ*tol-pal* cells accumulate dnGs and chain, effects that are reversed by overexpression of *digH* (19). In the same study, DigH was also shown to accumulate at septal regions and degrade dnGs in a purified system (19). Altogether, these observations indicate some role for DigH in processing septal PG. However, since no morphological phenotypes have been reported for *digH* deletion mutants, uncertainties surround the physiological role of DigH. Based on the aforementioned observations and the ΔLT-DigH connection, we sought to determine if DigH plays an active role in cell separation. To do this, we revisited the *digH* deletion phenotype. As shown in Fig. 4A, although deleting *digH* from wild-type cells produced no discernible effect on cell shape, deleting *digH* from ΔLT cells produced a potent synthetic effect; ΔLTΔ*digH* cells grew as extremely long chains, and individual cell units within the chains were morphologically less distinctive (i.e., septa were less constricted) and more distorted than those produced by the ΔLTΔ*prc*Δ*digH* mutant (compare ΔLTΔ*digH* derivatives in Fig. 3 to those in Fig. 4A). Like ΔLTΔ*prc*Δ*digH* cells, ΔLTΔ*digH* cells also grew poorly and flocculated (not shown). Since deleting *digH* increased chaining in ΔLT cells, we reasoned that overexpressing *digH* would decrease chaining in this dnG-accumulating strain background, but not in amidase-deficient (Δ*amiABC*) cells, which do not generate dnGs (15). Indeed, overexpressing *digH* restored rod shape to ΔLT (Fig. 4B), but not Δ*amiABC* cells (Fig. S3). Flow cytometry analysis also showed that ΔLT/p*digH* cells were closer in size to wild-type derivatives, though not completely (Fig. 4C). This finding further supports the notion that shape suppression in ΔLTΔ*prc* cells is fully achieved by the activities of multiple Prc-regulated hydrolases. Thus, our results strongly indicate that DigH plays an active role in cell separation by degrading dnGs. The synthetic phenotype of the ΔLTΔ*digH* mutant also indicates that the physiological function of DigH overlaps with one or more LTs.

During the course of these studies, we also examined the effect of deleting *digH* in cells disrupted for the Tol-Pal complex since these cells also accumulate dnGs (19) and grow as chains of unseparated cells (32–36). Interestingly, deleting *digH* did not alter the shape of a Δ*pal* mutant when grown in LB or LB lacking NaCl (Fig. S4, compare Δ*pal*Δ*digH* to Δ*pal* cells), presumably because the full complement of LTs is active in this strain background. This finding further supports the likelihood that the physiological function of DigH overlaps with one or more LTs.

### Evidence that dnGs are enriched in ΔLTΔ*digH* cells

**Fig 5:**
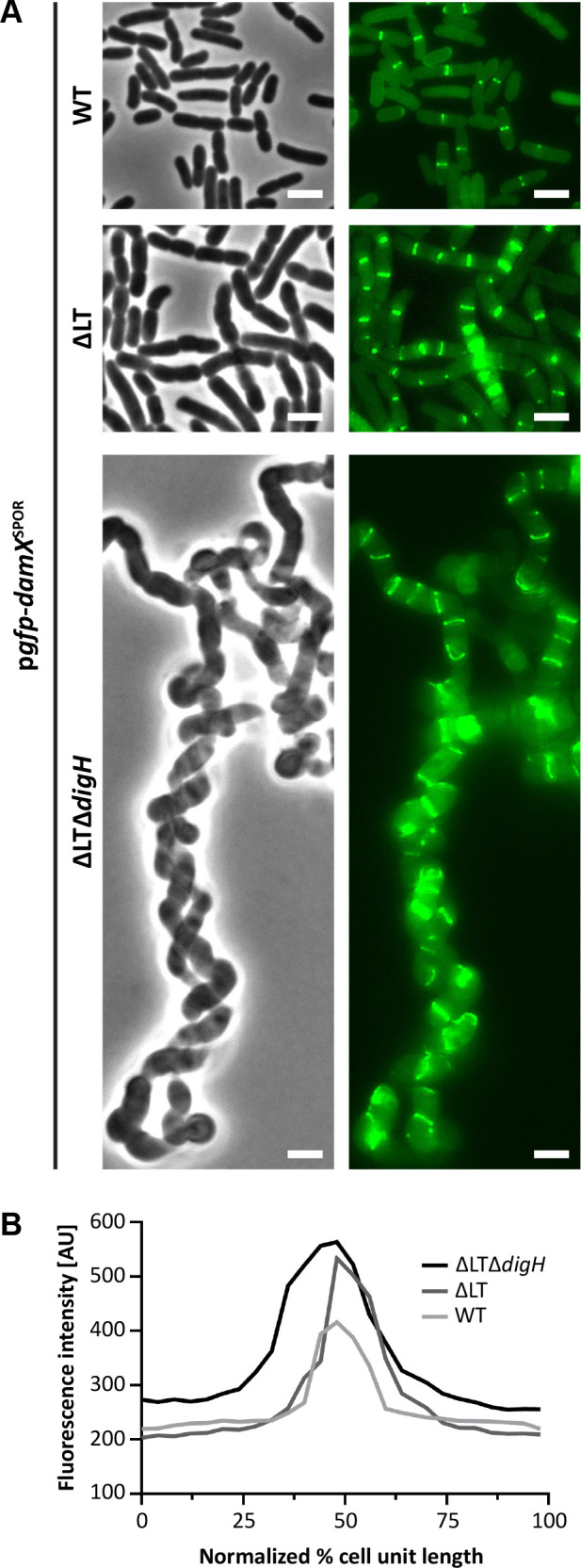
SPOR domain localization is enhanced in ΔLT cells lacking DigH. (**A**) Micrographs of cells with the indicated genotypes expressing a GFP fusion to the SPOR domain of DamX (preferentially binds dnGs). Overnight cultures were diluted 1:50 in LB (without induction), grown at 37°C to an OD_600_ ~0.4, and imaged by phase-contrast and fluorescence microscopy. We note that ΔLTΔ*digH*/p*gfp-damX*^SPOR^ cells were imaged after overnight growth. Bar, 3 µm. Data are representative of two independent experiments. The strains shown are MAJ1909 (WT/p*gfp-damX*^SPOR^), MAJ1864 (ΔLT/p*gfp-damX*^SPOR^), and MAJ1880 (ΔLTΔ*digH*/p*gfp-damX*^SPOR^). (**B**) Average fluorescence profiles plotted against normalized cell lengths for cells in panel A. Fluorescence intensity was measured across 20 septa and is expressed in arbitrary units [AU].

Since deleting *digH* increased chaining in ΔLT cells, we reasoned that dnGs were enriched in ΔLTΔ*digH* cells. To visualize dnGs, we expressed a GFP fusion to the SPOR domain from DamX (GFP-DamX^SPOR^) in wild-type, ΔLT, and ΔLTΔ*digH* cells (37). We note that SPOR domains localize to septal regions by binding specifically to dnGs (15, 38, 39). As shown in Fig. 5A, GFP-DamX^SPOR^ localized sharply to sites of constriction in wild-type, ΔLT, and ΔLTΔ*digH* cells. As expected, fluorescence intensity was higher in ΔLT derivatives than in wild-type cells (Fig. 5B). While peak fluorescence intensity was similar in ΔLT and ΔLTΔ*digH* cells, GFP-DamX^SPOR^ labeling was generally more diffuse in ΔLTΔ*digH* cells (Fig. 5B). This finding indicates that dnGs are even more enriched in this strain background and further suggests that DigH plays an active role in degrading dnGs at the septum. In summary, our findings indicate that DigH and LTs work together to degrade dnGs at the septum to support cell division in *E. coli*.

## DISCUSSION

During bacterial cell division, constriction requires splitting of septal peptidoglycan (PG) at the midcell by PG hydrolases. In bacteria like *E. coli*, *N*-acetylmuramyl-L-alanine amidases promote cell separation by cleaving peptide linkages from the glycan backbone of septal PG, which generate peptide-free (denuded) glycans (8, 9). Denuded glycans (dnGs) are then cleaved by lytic transglycosylases (LTs) (15, 17, 18). Mutants lacking multiple LTs accumulate dnGs at septal regions and grow as chains of unseparated cells (15). The DigH glycosyl hydrolase also degrades dnGs (19). However, since no morphological phenotypes have been described for a *digH* deletion mutant, the precise role for this enzyme remains uncertain. Here, we show that DigH is critical for cell separation in *E. coli* cells that accumulate dnGs. This finding clearly demonstrates that DigH plays an active role in remodeling the PG layer at the septum. While we show how cells containing DigH can evolve to suppress defects in LT activity, whether systems without DigH can also evolve workarounds remains to be determined. Altogether, findings from this study help provide a clearer understanding of PG remodeling during cell division.

### Deciphering the functional importance of cell wall hydrolases

Bacteria like *E. coli* usually encode many cell wall hydrolases with overlapping roles (3, 40, 41). This apparent functional redundancy exists for virtually every class of cell wall hydrolase, such that multiple deletions are often required to provoke cell wall defects (10, 11, 13, 14, 17, 42) and, in some cases (as we show for DigH), demonstrate physiological importance (43, 44). Since deleting *digH* blocks division in ΔLT cells, this strongly suggests that DigH activity is overlapped not only enzymatically but also spatially (midcell) and temporally (septation) by one or more LTs. To that end, MltA and MltE (which are deleted in ΔLT cells) have been shown to degrade dnGs (45–47). More generally, the multiplicity of cell wall hydrolases has led to a fundamental question: how many cell wall hydrolases are really necessary for cells to thrive? This question was partially answered with respect to the gram-positive bacterium *Bacillus subtilis*, which was shown to require only 1 out of 42 cell wall hydrolases for growth in rich media (7). Whether the cell wall hydrolase repertoire can be reduced to such an extent in gram-negative bacteria like *E. coli* remains to be seen.

### Spatial organization of peptidoglycan at the septum

**Fig 6:**
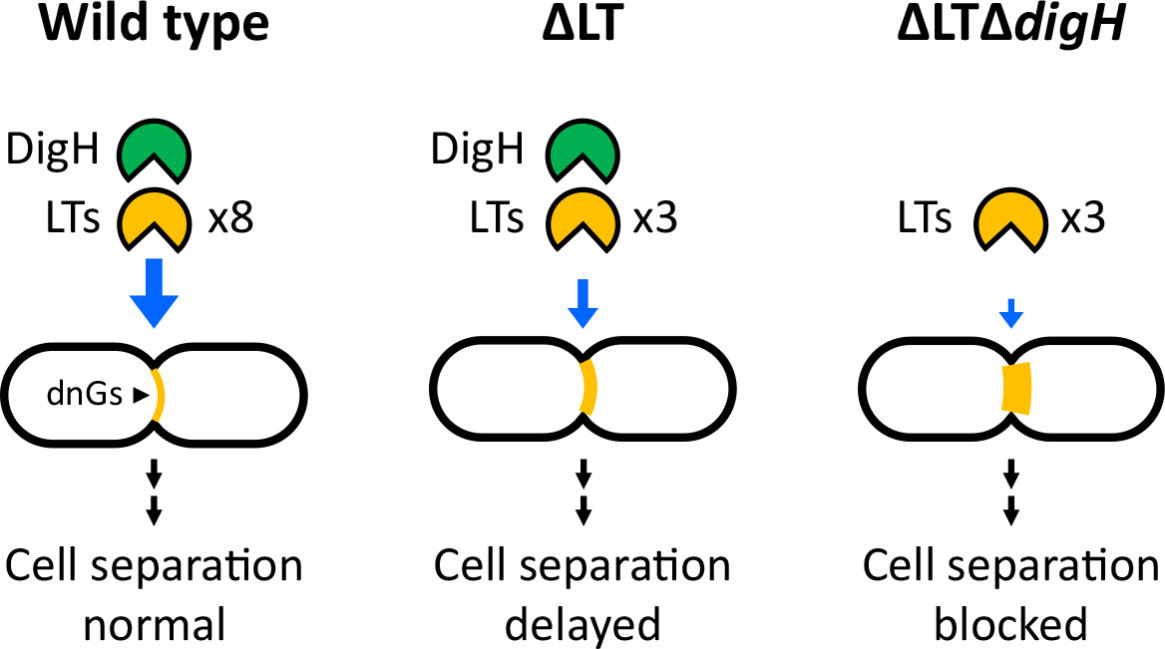
Model for denuded glycan degradation in *E. coli*. During bacterial cell division, denuded glycans (dnGs) are generated at the septum when amidases cleave PG cross-links from *N*-acetylmuramic acid residues. In the gram-negative bacterium *Escherichia coli*, dnGs are then degraded by a combination of LTs and the DigH glycosyl hydrolase. In wild-type cells, dnGs that are shared between the developing daughter cells are quickly degraded, and cells divide normally. In ΔLT cells (Δ*mltACDE*Δ*slt*Δ*rlpA*), dnGs accumulate, and cells are delayed for cell separation. When *digH* is deleted from ΔLT cells, dnGs accumulate further and cell separation is virtually blocked.

PG from rod-shaped bacteria like *E. coli* consists of glycan chains that are, on average, circumferentially ordered (i.e., arranged perpendicular to the long axis of the cell) (48). However, as *E. coli* loses its rod shape, the PG layer becomes increasingly disordered as glycan chains insert at more angles (49). At the septum, PG disorder likely involves glycans crossing the division plane such that the activity of any one LT becomes increasingly important for cell separation as more and more glycan bridges form between the developing daughter cells (Fig. 6). Since individual cell units in the ΔLT mutant are misshapen (Fig. 1), it is likely that glycan bridges form at a higher frequency in this strain background. If so, this could help explain why deleting *digH*, which specifically cleaves dnGs at the septum (15), blocks cell division in ΔLT cells. Glycan bridges have also been proposed to explain why deleting *rlpA*, which also cleaves dnGs at the septum (15, 18), induces a lethal chaining phenotype in a *Vibrio cholerae* mutant lacking six LTs (17). Thus, one possibility for why bacteria like *E. coli* and *V. cholerae* encode a multiplicity of enzymes that degrade PG glycan strands may be to safeguard against factors that disrupt PG orientation.

### Glycan degradation and peptidoglycan recycling

During growth and division, LTs cleave the PG sacculus, releasing muropeptides containing 1,6-anhydroMurNAc residues (5). DigH can also release anhydromuropeptides by cleaving the termini of dnGs, which are capped by 1,6-anhydroMurNAc ends (19). In *E. coli*, the majority of these anhydromuropeptides are transported by the AmpG permease back into the cell where they are broken down for reuse (50). Since LT activity is limiting in ΔLT cells, deleting *digH* is expected to further decrease the amount of PG that is recycled in this strain background. If defects in PG recycling contribute to the shape defect of ΔLT cells, this could help explain why deleting *digH* further distorts the morphology of this strain background.

### Chromosomal rearrangements drive genomic plasticity and evolution

Large-scale chromosomal rearrangements, including inversions, duplications, and deletions, play important roles in the evolutionary trajectory of organisms. In bacteria, large-scale rearrangements have been observed in gram-positive (51, 52) and gram-negative (53–57) bacteria and are often driven by recombination between homologous sequences in ribosomal operons (58), transposons, insertion sequence (IS) elements, and prophages (28). With the exception of cells exposed to mutagens, chromosomal rearrangements occur spontaneously and lead to genomic and, consequently, phenotypic changes that can be beneficial, as observed for ΔLT^sup^ cells and for other systems (59–61). Though we cannot fully explain the mechanism underlying the deletion in ΔLT^sup^ cells, the homologous sequences detected in *prc*, *yebZ*, and *manX* (Fig. S1) suggest the deletion in ΔLT^sup^ cells was driven by homologous recombination. If so, this brings up an interesting question: why are repeated sequences found in *prc*? Since Prc negatively regulates DigH activity (19), one possibility is that the repeated sequences in *prc* function as a failsafe against LT inhibitors like Bulgecin A (62, 63). However, whether cells can rely solely on DigH activity for glycan strand degradation remains to be determined.

## MATERIALS AND METHODS

### General procedures

All strains, plasmids, and primers are listed in Tables S1 to S3, respectively. *E. coli* cells were cultured in LB Miller medium consisting of 1% tryptone, 0.5% yeast extract, and 1% NaCl (IBI Scientific). LB plates contained 1.5% agar (Difco). As needed, antibiotics were used at the following concentrations: 100 µg mL^−1^ ampicillin and 50 µg mL^−1^ kanamycin.

### Strain construction

*E. coli* MG1655 is the parent strain for this study. Genes were deleted by using a combination of lambda Red recombination (64) and P1 transduction. FRT-flanked kanamycin resistance markers were evicted by using FLP recombinase produced from pCP20 (65). Gene deletions were designed using sequences obtained from the EcoCyc database (66). All gene deletions were verified by PCR.

### Plasmid construction

pMAJ233 (P_204_::*digH*) is a plasmid that expresses *digH* and was constructed by amplifying *digH* from MG1655 DNA with primers P1440 and P1441. The 1,335 bp product was digested with EcoRI and HindIII and ligated to the same sites of pDSW204 (29). pMAJ240 (P_206_::*prc*) is a plasmid that expresses *prc* and was constructed by amplifying *prc* from MG1655 DNA with primers P1432 and P1433. The 2,064 bp product was digested with EcoRI and SmaI and ligated to the same sites of pDSW206 (29). Oligonucleotide primers were synthesized by Eurofins Genomics. Restriction enzymes were obtained from New England Biolabs. Plasmid constructs were verified by DNA sequencing at the UAMS DNA Sequencing Core Facility.

### Selection for ΔLT suppressors

Cultures of MAJ718 (ΔLT) were grown overnight in a New Brunswick Gyrotory Water Bath Shaker Model G76 temperature set at 37°C, speed at 5, and high heat. Overnight cultures were diluted 1 × 10^−5^ in the LB medium and plated on LB agar. Plates were incubated at 37°C overnight. ΔLT cells produced mucoid colonies, and ΔLT suppressors produced non-mucoid colonies.

### Colony phenotyping

Overnight cultures were diluted 1 × 10^−5^ in LB medium and plated on LB agar. Plates were incubated at 37°C overnight and imaged the next day by using an iPhone 13 Pro (Apple Inc.).

### Morphological analyses

Overnight cultures were diluted 1:2,000 in LB medium and grown at 37°C (unless noted otherwise) to an optical density at 600 (OD_600_) of ~0.4–0.5. Live cells were spotted onto 1% agarose pads and imaged by phase-contrast microscopy by using an Olympus DP23M monochrome camera coupled to an Olympus BX60 microscope. The ΔLTΔ*digH* mutant was imaged after overnight growth. Live cells were also used for flow cytometry and were prepared by pelleting 1 mL of cells (above), washing twice in room temperature phosphate-buffered saline (PBS, 137 mM NaCl, 3 mM KCl, 9 mM NaH_2_PO_4_, and 2 mM KH_2_PO_4_, pH 7.4), and diluting to an OD_600_ ~0.0.5. Cells were then analyzed by using the forward scatter detector in a BD LSRFortessa Flow Cytometer at the UAMS Flow Cytometry Core Facility.

### GFP-DamX^SPOR^ localization

Cells producing GFP fused to the SPOR domain of DamX were grown overnight at 37°C in the LB medium containing ampicillin, diluted 1:50 into the same medium, and grown for 2.5 h. Live cells were then spotted onto 1% agarose pads and imaged by phase-contrast and fluorescence microscopy. ΔLTΔ*digH*/p*gfp-damX*^SPOR^ cells were imaged after overnight growth. Line profiles were measured using the “line profile” tool in cellSens Dimension software version 4.2.1 (Olympus).

### Whole-genome sequencing

For Nanopore sequencing, genomic DNA was extracted and purified from overnight cultures of MAJ718 (ΔLT) and MAJ1075 (ΔLT^sup^) by using DNAzol (Molecular Research Center, Inc.) and the Genomic DNA Clean and Concentrator Kit (Zymo Research). Purified genomic DNA (~800 ng) was multiplexed by using the Rapid Barcoding Sequencing kit (SQK-RBK004; ONT, Oxford, UK). Sequencing libraries were then loaded onto a FLO-MIN106 R9.4.1 flow cell and sequenced by using an Oxford Nanopore MinION device under MinKnow software (ONT, Oxford, UK). Base calling and demultiplexing of barcodes were performed by using Guppy v6.5.7 (67). Reads longer than 200 bp with a quality score above 7 were extracted after 72 hours of the sequencing run for further analysis. The filtered reads from MAJ718 and MAJ1075 were mapped against the *E. coli* MG1655 reference genome (NCBI RefSeq assembly GCF_904425475.1) using Minimap2 version 2.17-r94 (68). Structural variation analysis was investigated using Sniffle tools version 2.2 (69). The selected regions of interest were visualized by using the Integrative Genomics Viewer software version 2.17.3 (70).

For Illumina sequencing, genomic DNA was extracted and purified from overnight cultures of MAJ718 (ΔLT) and MAJ1075 (ΔLT^sup^) by using the Monarch Genomic DNA Purification Kit (NEB). Purified genomic DNA (~600 ng) was then sequenced by SeqCenter (Pittsburgh, PA) using 151 bp paired-end reads on an Illumina NovaSeq 6000 sequencer. Processing and analysis of sequencing data were performed using BV-BRC (71). Paired FASTQ files were trimmed and aligned by using FastQ Utilities. Sequences were analyzed by variant analysis using the *E. coli* MG1655 reference genome (NCBI RefSeq assembly RCF_904425475.1). Deletions were confirmed via the genome browser.

## Data Availability

All Nanopore sequencing reads have been deposited in the NCBI database under SRA accession numbers SRR32271206 (MAJ1075) and SRR32271207 (MAJ718). All Illumina sequencing reads have been deposited in the NCBI database under SRA accession numbers SRR32429794 (MAJ1075) and SRR32429795 (MAJ718). The BioProject accession number is PRJNA1221016. The Python code used to bin line profiling data by length segments is available from the corresponding author upon request.

## SUPPLEMENTAL MATERIAL

Supplemental tables and figures jb.00068-25-s0001.pdf Tables S1 to S3 and Fig. S1 to S4.

ASM does not own the copyrights to Supplemental Material that may be linked to, or accessed through, an article. The authors have granted ASM a non-exclusive, world-wide license to publish the Supplemental Material files. Please contact the corresponding author directly for reuse.
