# Supplementary material for: The DigH glycosyl hydrolase is conditionally required for daughter cell separation in Escherichia coli
Source: J Bacteriol. 2025 Jun 10;207(7):e00068-25. doi: 10.1128/jb.00068-25 (PMC12288456; doi:10.1128/jb.00068-25)
Supplement: Supplemental tables and figures — Tables S1 to S3 and Fig. S1 to S4. [file jb.00068-25-s0001.pdf]

Supplemental information for:

The DigH glycosyl hydrolase is conditionally required for daughter cell separation in *Escherichia coli*

Joseph C. Bryant[1], Emily J. Robbs[1], Alongkorn Kurilung[2], Brittney A. Dinkel[3], Intawat Nookaew[2], and Matthew A. Jorgenson[1*]

1. Department of Microbiology and Immunology, University of Arkansas for Medical Sciences, Little Rock, AR, USA
2. Department of Biomedical Informatics, University of Arkansas for Medical Sciences, Little Rock, AR, USA
3. School of Science, Buena Vista University, Storm Lake, IA, USA

*Corresponding author

4301 West Markham St. / Biomed I, Room 511 / Little Rock, AR 72205

E-mail: majorgenson@uams.edu

Phone: 501-686-7706

Table S1. Strains used in this study

Strain	Relevant features	Source
MAJ1	MG1655 F^- λ^- *rph*-1 *ilvG wbbL*::IS*5* (is wild-type)	Lab collection
MAJ280	MAJ1 Δ*mepS*::*kan*	This study
MAJ286	MAJ1/pDSW204	(1)
MAJ305	MAJ1 Δ*nlpI*::*kan*	This study
MAJ718	MG1655 Δ*rlpA*::*frt* Δ*mltA*::*frt* Δ*slt*::*frt* Δ*mltD*::*frt* Δ*mltE*::*frt* Δ*mltC*::*frt* (is EC3708)	(2)
MAJ1075	MAJ718 Δ(*manY*-'*yebZ*)	This study
MAJ1780	MAJ718 Δ*nlpI*::*kan*	This study
MAJ1794	MAJ1 Δ*prc*::*kan*	This study
MAJ1795	MAJ718 Δ*prc*::*kan*	This study
MAJ1801	MAJ718 Δ*prc*::*frt*	This study
MAJ1807	MAJ1801 Δ*mepS*::*kan*	This study
MAJ1808	MAJ718/pDSW204	This study
MAJ1812	MAJ1801 Δ*digH*::*kan*	This study
MAJ1813	MAJ1801 Δ*mltG*::*kan*	This study
MAJ1814	MAJ1 Δ*digH*::*kan*	This study
MAJ1815	MAJ718 Δ*digH*::*kan*	This study
MAJ1830	MAJ718/pMAJ233	This study
MAJ1833	MAJ1 Δ*mltB*::*kan*	This study
MAJ1834	MAJ1801 Δ*mltB*::*kan*	This study
MAJ1841	MAJ1 Δ*pal*::*kan*	This study
MAJ1854	MAJ1 Δ*mltG*::*kan*	This study
MAJ1864	MAJ718/pDSW997	This study
MAJ1880	MAJ1815/pDSW997	This study
MAJ1895	MAJ1075/pDSW206	This study
MAJ1897	MAJ1075/pMAJ240	This study
MAJ1909	MAJ1/pDSW997	This study
MAJ1929	MAJ1814 Δ*digH*::*frt*	This study
MAJ1931	MAJ1929 Δ*pal*::*kan*	This study
MAJ1934	MAJ1/pMAJ233	This study
MAJ1960	CS109 *amiB yje25*::*res amiA*::*cam amiC*::*kan*/pDSW204	This study
MAJ1961	CS109 *amiB yje25*::*res amiA*::*cam amiC*::*kan*/pMAJ233	This study
**Additional *E. coli* strains used as donors for P1 transduction**		
JW0731-1	BW25113 Δ*pal*::*kan*	(3)
JW1083-1	BW25113 Δ*mltG*::*kan*	(3)
JW1486-1	BW25113 Δ*digH*::*kan*	(3)
JW1819-2	BW25113 Δ*prc*::*kan*	(3)
JW2671-1	BW25113 Δ*mltB*::*kan*	(3)

Table S2. Plasmids used in this study

Plasmid	Relevant genotype or characteristics	Replication origin	Source or reference
pCP20	λ_{PR}::*flp* λcI857 *bla cat* Rep(Ts)	pSC101	(4)
pDSW204	P_{204} *lacI*^{q} *bla*	pBR	(5)
pDSW206	P_{206} *lacI*^{q} *bla*	pBR	(5)
pDSW997	P_{204}::^{TT}*gfp-damX*^{SPOR}(338–428)	pBR	(6)
pKD13	*bla frt-aph-frt*	R6Kγ	(7)
pKD46	P_{araB}::*gam-bet-exo bla* Rep(Ts)	pSC101	(7)
pMAJ233[a]	P_{204}::*digH*	pBR	This study
pMAJ240[b]	P_{206}::*prc*	pBR	This study

[a]Derivative of pDSW204

[b]Derivative of pDSW206

Table S3. Primers used in this study

Primer	Sequence	Purpose
P142	TATTTGTCGTTAAGGACTTCAAGGGAAAACAAACAACATGATTCCGGGGATCCGTCGACC	Δ*mepS*
P143	ATTGCATCCAAACGGTTTATTAGCTGCGGCTGAGAACCCGTGTAGGCTGGAGCTGCTTCG	Δ*mepS*
P148	AGGACGTTCATTCAACCGTGGTCTTCGGGAGTGGGAAATGTGTAGGCTGGAGCTGCTTCG	Δ*nlpI*
P149	GGGCTGATGTGTACGTCAGCTATTGCTGGTCCGATTCTGCATTCCGGGGATCCGTCGACC	Δ*nlpI*
P1432	CAGGAATTCAACATGTTTTTTAGGCTTACCGCG	pMAJ240
P1433	TTGCCCGGGTTACTTGACGGGAGCGGGTTG	pMAJ240
P1440	CAAGAATTCGATATCTGCTCCCGAAACAAG	pMAJ233
P1441	CTGAAGCTTTTAACTGCCCCAGCGACTTTG	pMAJ233

[a]All primer sequences are written 5'→3'. Restrictions sites are underlined.

References

1. Kay EJ, Dooda MK, Bryant JC, Reid AJ, Wren BW, Troutman JM, Jorgenson MA. 2024. **Engineering *Escherichia coli* for increased Und-P availability leads to material improvements in glycan expression technology**. *Microb Cell Fact* 23:72.
2. Yahashiri A, Jorgenson MA, Weiss DS. 2015. **Bacterial SPOR domains are recruited to septal peptidoglycan by binding to glycan strands that lack stem peptides**. *Proc Natl Acad Sci USA* 112:11347-52.
3. Baba T, Ara T, Hasegawa M, Takai Y, Okumura Y, Baba M, Datsenko KA, Tomita M, Wanner BL, Mori H. 2006. **Construction of *Escherichia coli* K-12 in-frame, single-gene knockout mutants: the Keio collection**. *Mol Syst Biol* 2:2006 0008.
4. Cherepanov PP, Wackernagel W. 1995. **Gene disruption in *Escherichia coli*: TcR and KmR cassettes with the option of Flp-catalyzed excision of the antibiotic-resistance determinant**. *Gene* 158:9-14.
5. Weiss DS, Chen JC, Ghigo JM, Boyd D, Beckwith J. 1999. **Localization of FtsI (PBP3) to the septal ring requires its membrane anchor, the Z ring, FtsA, FtsQ, and FtsL**. *J Bacteriol* 181:508-20.
6. Arends SJ, Williams K, Scott RJ, Rolong S, Popham DL, Weiss DS. 2010. **Discovery and characterization of three new *Escherichia coli* septal ring proteins that contain a SPOR domain: DamX, DedD, and RlpA**. *J Bacteriol* 192:242-55.
7. Datsenko KA, Wanner BL. 2000. **One-step inactivation of chromosomal genes in *Escherichia coli* K-12 using PCR products**. *Proc Natl Acad Sci USA* 97:6640-5.

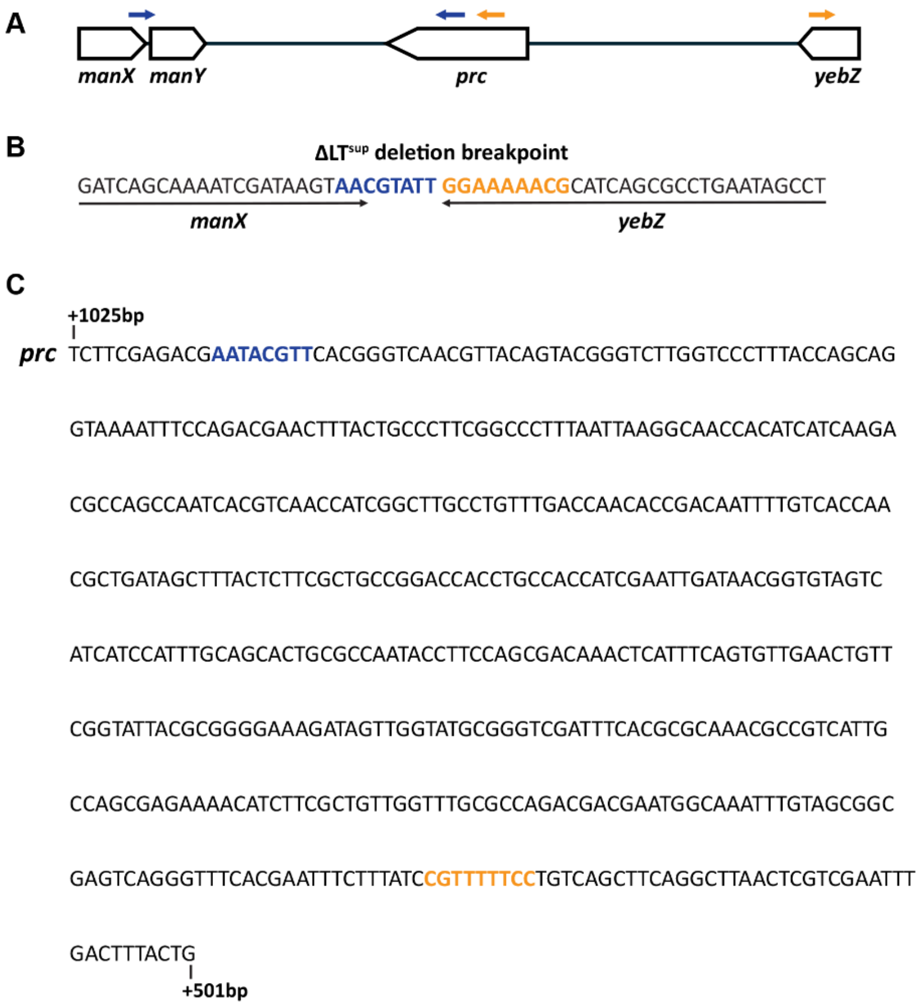

Figure S1. Repeated sequences in the LT^{sup} breakpoint and *prc*. (A) Genomic representation showing a subset of genes in the *manX*→*yebZ* region in *E. coli* MG1655 cells. The direction of inverted repeats (arrows) is given relative to the orientation of *prc*. (B) Nucleotide sequences surrounding the ΔLT^{sup} deletion breakpoint. Sequence upstream of the breakpoint is shaded blue, while the sequence downstream of the breakpoint is shaded orange. Sequence repeated in *prc* is bolded. (C) Partial *prc* sequence. Nucleotide positions relative to the start site are indicated above the sequence. Bolded sequences correspond to sequences found at the end of *manX*, as well as in *yebZ*.

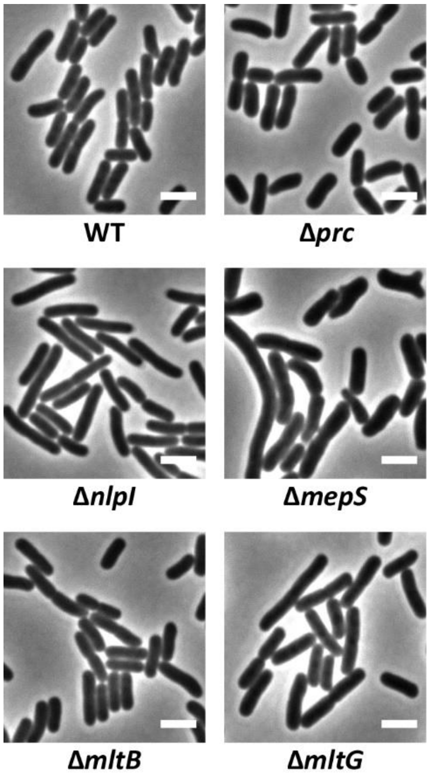

Figure S2. Micrographs of individual PG hydrolase mutants. Cells with the indicated genotypes were grown in LB at 37°C until the culture reached an OD_{600} of ~0.4-0.5. The cells were then photographed by phase-contrast microscopy. Bar, 3 µm. The strains shown are MAJ1 (WT), MAJ1794 (Δ*prc*), MAJ305 (Δ*nlpI*), MAJ280 (Δ*mepS*), MAJ1833 (Δ*mltB*), and MAJ1854 (Δ*mltG*).

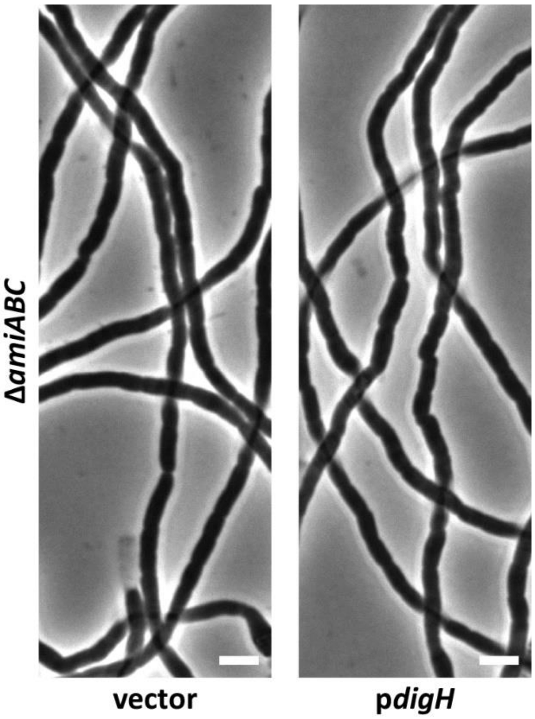

Figure S3. Overexpressing *digH* does not reverse chaining in Δ*amiABC* cells.

Micrographs of Δ*amiABC* cells containing vector or p*digH*. Cells were diluted 1:200 in LB containing 25 µM IPTG and grown at 37°C until the culture reached an OD_{600} of ~0.4-0.5. The cells were then photographed by phase-contrast microscopy. Bar, 3 µm. The strains shown are MAJ1960 (Δ*amiABC*/vector) and MAJ1961 (Δ*amiABC*/p*digh*)

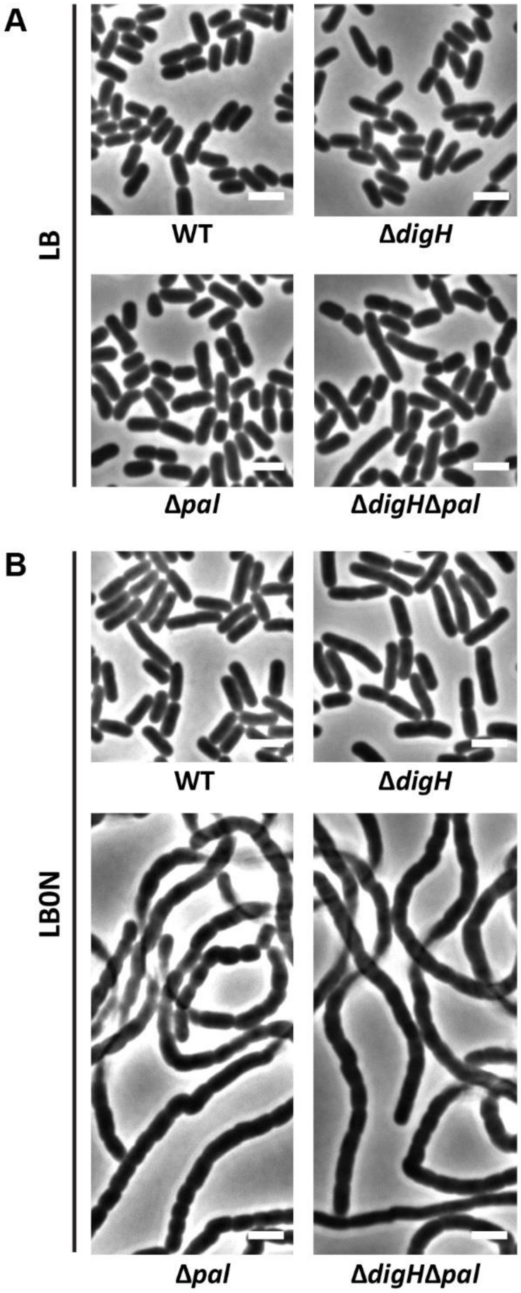

Figure S4. Deleting *digH* does not exacerbate the shape defect of Δ*pal* cells. Cells with the indicated genotypes were diluted 1:2,000 in LB or 1:200 in LB0N and grown at 42°C until the culture reached an OD_{600} of ~0.4-0.5. The cells were then photographed by phase-contrast microscopy. Bar, 3 µm. The strains shown are MAJ1 (WT), MAJ1814 (Δ*digH*), MAJ1841 (Δ*pal*),and MAJ1931 (Δ*digH*Δ*pal*).
